# Complete Genome Sequence of *Myxococcus* Phage Mx4

**DOI:** 10.1128/MRA.00953-21

**Published:** 2021-10-21

**Authors:** Sébastien Wielgoss

**Affiliations:** a Institute of Integrative Biology, Department of Environmental Systems Science, ETH Zürich, Zürich, Switzerland; DOE Joint Genome Institute

## Abstract

Myxococcus xanthus is a bacterial model in microbial developmental biology and social evolution. Here, I present the 57.0-kb circular genomic sequence of the wild-type *Myxococcus* phage Mx4, with a GC content of 70.1%. Annotation predicted 97 protein-coding genes. Head-neck-tail protein classification assigns Mx4 to the tailed, Mu-like members of the family *Myoviridae* of group type 1 (cluster 8).

## ANNOUNCEMENT

**FIG 1:**
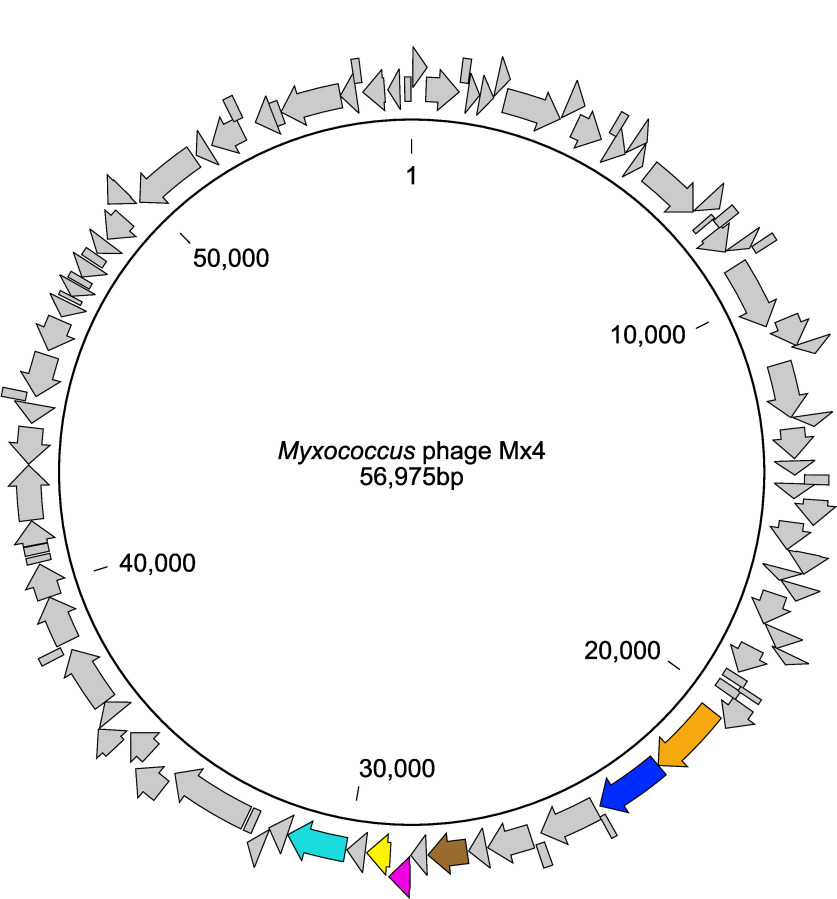
Circular genome of *Myxococcus* phage Mx4, containing 97 protein-coding genes (arrows). The colors are based on the protein classification in Virfam (26): orange, large terminase protein (UAW08042); blue, portal protein (UAW08043); brown, major capsid protein (UAW08049); magenta, adaptor protein (UAW08051); yellow, neck protein (UAW08052); mint, tail sheath (UAW08054). The inner ring labels depict the position in base pairs.

Myxococcus xanthus is a predatory deltaproteobacterium in soil (1). It is a well-studied model organism in developmental biology and social evolution (2), and various aspects of its multicellular life cycle have been studied in molecular detail (3–14). Bacteriophages of *Myxococcus* have been isolated and used experimentally (15); however, a complete genome sequence is available for only one, Mx8 (unpublished; GenBank accession number AF396866). Here, I present the whole-genome sequence of the lytic wild-type *Myxococcus* phage Mx4. The sample traces back to the wild-type isolate from the 1970s, originally derived by infecting the susceptible laboratory strain Myxococcus xanthus DZ1 with resuspended and filtered soil and manure of mixed origins (16). Mx4 was kept in MOPS (3-morpholinopropanesulfonic acid)-based phage buffer at 4°C and was frequently passaged in the aforementioned host (17). A fresh high-titer stock was prepared from one clear plaque at 32°C in the Myxococcus xanthus host strain DZ1, following standard methods for lytic myxophage propagation (17, 18). To extract genomic DNA, I infected strain DZ1 (5 × 10^8^ CFU/ml) with Mx4 at a multiplicity of infection (MOI) of ∼2 in modified CTT broth (8 mM MgSO_4_, 10 mM Tris [pH 8.0], 20 g/liter Casitone, 1 mM KPO_4_, and 1 mM CaCl_2_) for 60 min without agitation at room temperature prior to setting up a double agar overlay (18). After 2 days of incubation (32°C, 90%rH), the confluently lysed DZ1 lawn was covered with 2 ml genome buffer B1 (Qiagen) and kept at 4°C for 24 h to dissolve free phage particles. Genomic DNA from this suspension, as well as a pellet of the uninfected DZ1 control, were purified using Qiagen’s blood and tissue kit following the manufacturer’s recommendations. DNA was sheared to ∼350-bp lengths using a multifunctional bioprocessor (EpiSonic) and prepared for Illumina short-read sequencing in 150-bp paired-end mode on the same flow cell lane on a HiSeq 4000 machine using the NEBNext Ultra DNA library prep kit. The samples were demultiplexed using Illumina’s bcl2fastq v2.20.0. Reads from both treatments were quality checked using fastqc v0.11.8 (19) and trimmed to lengths of >80 bp using Trimmomatic v0.32 (20) with default parameters, leaving ∼70% of the sequence information (Table 1). To remove any host-derived reads from the Mx4 infection treatment, I devised the following approach. The trimmed DZ1 control reads were *de novo* assembled using SPAdes v3.11.1 (21) with “-k 21,33,55,77,99,127 --careful” and the resulting contigs served as a reference to which trimmed reads of the Mx4 infection treatment were mapped using breseq v0.28.1 (22). The 175,376 reads (average length, ∼145.1 bp) of the Mx4 infection treatment (∼29%) (Table 1) that did not match the DZ1 control contigs, i.e., the enriched Mx4 reads, were assembled using SPAdes v3.11.1 (21), with the same parameters as above. This procedure resulted in a total of 22 contigs, and the removal of low coverage contigs (k-mer coverage, <10) and manual curation led to a single contig with overlapping ends, which indicates a circular genome. The completed Mx4 genome contained 56,975 bp (Fig. 1) with ∼458-fold coverage, in line with the predicted size range for Mx4 (15). The genome’s GC content, ∼70.1%, precisely matched the early predictions based on buoyant density measurements (23). RASTtk-enabled gene annotation with the Virus option in PATRIC (24, 25) predicted 97 protein-coding genes. The coding density was ∼95.5%. Head-neck-tail protein classification in Virfam (26) assigned Mx4 to the Mu-like tailed viruses of the *Myoviridae* group type 1 (cluster 8), in line with the physical characterization of Mx4 morphology (23).

**TABLE 1:** Illumina sequencing read statistics

Treatment	SRA library name	SRA accession no.	No. of raw reads	No. of trimmed reads	No. of unmatched reads^*a*^	Size of raw sequences (Mbp)^*b*^	Size of trimmed sequences (Mbp)^*b*^	Size of unmatched sequences^*a*^ (Mbp)^*b*^
DZ1	CONTROL	SRR15834097	8,772,050	6,038,535		1,315.0	870.4	
Mx4 infection	MIX	SRR15834096	838,202	604,983		125.7	87.7	
Enriched Mx4^*a*^	UNMATCHED	SRR15834095			175,376			25.4

a Unmatched reads after mapping the MIX reads against the assembled CONTROL contigs.

b Mbp, million base pairs.

### Data availability.

The whole-genome sequence of *Myxococcus* phage Mx4 was deposited in DDBJ/ENA/GenBank under accession number OK085710. The raw sequencing reads were deposited in the Sequence Read Archive (SRA) under accession numbers SRR15834095 (phage), SRR15834096 (infected host), and SRR15834097 (uninfected host).
